# ONL1204 for the Treatment of Geographic Atrophy: Phase Ib Study Evaluating Safety, Tolerability, and Efficacy

**DOI:** 10.1016/j.xops.2025.100954

**Published:** 2025-10-03

**Authors:** David M. Kleinman, Charles C. Wykoff, Durga S. Borkar, Robyn H. Guymer, Andrew J. Kocab, Lindsay Puscas, Stephanie C. Wietholter, Lori L. Kesteloot, David N. Zacks

**Affiliations:** 1 ONL Therapeutics, Ann Arbor, Michigan; 2 Flaum Eye Institute, University of Rochester, Rochester, New York; 3 Retina Consultants of Texas, Bellaire, Texas; 4 Duke Eye Center, Durham, North Carolina; 5 Centre for Eye Research in Australia, Royal Victorian Eye and Ear Hospital, The University of Melbourne, East Melbourne, Australia

**Keywords:** Geographic atrophy, Lesion growth, Visual acuity, Dry age related macular degeneration

## Abstract

**Purpose:**

To evaluate the safety and tolerability of ONL1204, a novel, small peptide inhibitor of the fragment apoptosis stimulator receptor, for the treatment of patients with geographic atrophy (GA).

**Design:**

A phase Ib multicenter study involving a dose-escalation/open-label (DE/OL) component and a randomized, double-masked, sham-controlled natural history/treatment (NHS/T) component.

**Participants:**

Patients aged ≥55 years with GA secondary to age-related macular degeneration.

**Methods:**

Dose-escalation/OL patients received a single intravitreal injection of either 50 μg, 100 μg, or 200 μg of ONL1204 and were followed for 24 weeks. Participants in the NHS/T component were randomized (1:1:1) to either 50 μg or 200 μg of ONL1204 or sham injection, after a 24-week NHS phase. Two injections were administered 12 weeks apart, and patients were observed for an additional 12 weeks.

**Main Outcome Measures:**

The primary endpoint was safety, assessed by monitoring adverse events (AEs), ophthalmic examination, electrophysiology, fundus photography, fundus autofluorescence, and OCT. Additional endpoints included measurement of GA lesion area and best-corrected visual acuity.

**Results:**

Six patients were enrolled in the DE/OL component and 22 patients in the NHS/T component. All patients in the DE/OL component completed the study with no major safety findings or dose-limiting toxicities. Seventeen patients were randomized in the NHS/T component, with 15 patients completing the study. All ophthalmic AEs were mild or moderate in severity. ONL1204 demonstrated a favorable effect on GA lesion growth, with numerically favorable slower lesion growth compared with the fellow eye in the DE/OL component at 6 months and a numerically slower growth rate (mean difference [standard error of the mean] of –0.524 mm^2^ [0.39]; *P* = 0.202) in the 200 μg ONL1204 group compared with the sham group in the treatment phase of the NHS/T component. There were no changes in the treatment group with respect to visual acuity suggestive of any safety issues.

**Conclusions:**

ONL1204 was safe and well tolerated at all evaluated doses, with the potential to reduce GA lesion growth and improve vision. These results support further evaluation of ONL1204 in patients with GA.

**Financial Disclosure(s):**

Proprietary or commercial disclosure may be found in the Footnotes and Disclosures at the end of this article.

Age-related macular degeneration (AMD) is a leading cause of severe irreversible vision loss globally in individuals over the age of 55 years. The progressive loss of central vision limits activities such as reading, driving, and other tasks requiring fine vision, and late stages of the condition often render patients legally blind.^1^, ^2^, ^3^

Age-related macular degeneration can be classified according to the Beckman classification terminology, with early and intermediate AMD defined by the presence of drusen.^4^ There are 2 late-stage complications of AMD, neovascular AMD and geographic atrophy (GA). Pseudodrusen, deposits between the retinal pigment epithelium (RPE) and photoreceptors, are considered a risk factor for AMD progression.^5^

The pathophysiology of AMD is complex and poorly understood. Geographic atrophy is associated with RPE and photoreceptor cell death. While there are several biochemical pathways implicated in this cell death, there is strong evidence to suggest that the inflammatory pathway is involved (Fleckenstein et al).^6^ Intrinsic and extrinsic factors can play a role in chronic activation of these pathways, including the complement system. While treatments for neovascular AMD have been available for nearly 2 decades, the US Food and Drug Administration has only recently approved 2 treatments specific for GA; both target the complement system. Pegcetacoplan (Apellis Pharmaceuticals) and avacincaptad pegol (Astellas Pharma) are delivered via intravitreal (IVT) injection and have demonstrated efficacy in slowing the progression of GA lesion area by inhibiting the complement cascade.^7^, ^8^, ^9^ These therapeutic agents are promising in an area with no other treatment options, but the beneficial effect is modest and associated with a high treatment burden of monthly or every other month IVT injections as well as safety considerations. Thus, there remains an unmet need for a more effective and durable treatment option for GA.

Activation of the fragment apoptosis stimulator (Fas) receptor, through binding of the Fas ligand, results in programmed cell death, or apoptosis. In normal retinal tissue, the expression of the Fas receptor is very limited. In contrast, the photoreceptors and areas of RPE around atrophic lesions have been identified as having elevated expression of the Fas receptor in patients with AMD.^10^^,^^11^ ONL1204 (ONL Therapeutics) is being developed as a novel inhibitor of both Fas-mediated cell death and the associated inflammatory response at sites upstream of currently approved agents.^12^ It is hypothesized that inhibiting Fas-mediated cell death in the setting of GA secondary to AMD reduces cell death, preserving RPE and photoreceptors, thereby reducing vision loss in AMD. The purpose of this study is to evaluate the safety and tolerability of ONL1204 delivered at 2 different dose levels via IVT injection in patients with GA secondary to AMD. In addition, secondary efficacy endpoints were also utilized to gain insights into the required dose and frequency of administration for the treatment of GA.

## Methods

This was a phase Ib multicenter study. Patients were enrolled at 9 sites in Australia and New Zealand. The study was approved by Independent Ethics Committees. All participants provided written informed consent, and the study was conducted in accordance with the tenets of the Declaration of Helsinki, in compliance with the International Conference on Harmonization E6 guideline for Good Clinical Practice and all applicable guidelines. This study was initiated in July 2021, and the last patient completed the study in February 2024. The study was conducted during the period in which the coronavirus disease 2019 pandemic was occurring in the study region (Australia and New Zealand). Due to the pandemic, enrollment was protracted, and the duration of participation was prolonged for some patients. The study was registered through ClinicalTrials.gov (https://clinicaltrials.gov/study/NCT04744662; ID: NCT04744662).

**Figure 1:**
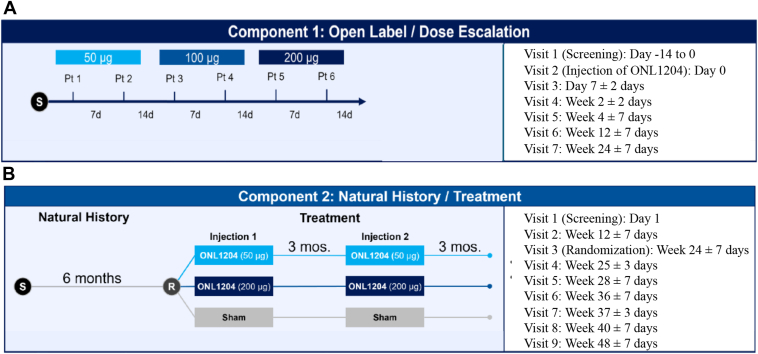
ONL1204 GA-001 study design for the open label/dose escalation component **(A)** and the natural history/treatment component **(B)**. d = days; mos = months; Pt = patient; R = randomization; S = screening.

The overall design of this phase Ib study involved 2 components. Component 1 evaluated 3 different doses of ONL1204 Ophthalmic Solution (ONL1204; 50 μg, 100 μg, and 200 μg) using a single-injection dose-escalation/open-label (DE/OL) design (Fig 1A). Component 2 utilized a natural history/treatment (NHS/T), which allowed for an initial observation period of 24 weeks, followed by randomization of patients to 2 treatments of either a 50 μg or 200 μg dose of ONL1204, or sham treatment, 3 months apart (Fig 1B). The DE/OL component of the trial was included because this study was the first to evaluate ONL1204 in a chronic ocular disease and without a pars plana vitrectomy after its intraocular delivery, as was utilized in a clinical trial of ONL1204 in rhegmatogenous retinal detachment (https://clinicaltrials.gov/study/NCT03780972; ID: NCT03780972).

### Patient Eligibility Criteria

The full list of inclusion/exclusion criteria is presented in the Supplemental Information (available at www.ophthalmologyscience.org). The main inclusion criteria for the DE/OL component required participants to be ≥55 years of age and have a diagnosis of GA secondary to AMD in the study eye. Participants were required to have a best-corrected visual acuity (BCVA) ranging from 20/100 to Counting Fingers (Snellen equivalent) as measured by the ETDRS chart. If GA was present in both eyes, the eye with the worse BCVA was selected as the study eye.

The main eligibility criteria for the NHS/T component of the study required participants to be ≥55 years of age and have a BCVA of 20/400 (Snellen equivalent) or better in both eyes. Participants in the NHS/T component were required to have bilateral GA secondary to AMD without a history of or current choroidal neovascularization in either eye as determined by the Doheny Image Reading Center and the investigator. Furthermore, the GA lesion in the study eye as determined by the reading center was to have the following characteristics: GA lesion size ≥1 disc area (equivalent to ≥2.5 mm^2^); if GA was multifocal, ≥1 focal lesion had to have a disc area of ≥1.25 mm^2^. In addition, the entire GA area had to have been visible within the standard (30 degree) fundus autofluorescence (FAF) field of view, and the presence of banded or diffuse hyperautofluorescence adjacent to the GA lesion was required.

Key exclusion criteria for both the DE/OL and NHS/T components of the study included atrophy secondary to conditions other than AMD, intraocular inflammation, or ocular/periocular infection in the study eye; a history of systemic use of pentosan polysulfate sodium; media opacity that would have limited visual acuity assessment or clinical visualization of the retina in the study eye; or any other ocular or systemic condition that, in the opinion of the investigator, made the patient unsuitable for treatment with an investigational agent or that would have compromised safety and efficacy assessments. Additionally, hyperautofluorescence adjacent to the GA lesion in the study eye that was focal only or previous IVT treatment, history of retinal surgery, or other retinal therapeutic procedures in the study eye were also exclusion criteria for the NHS/T component of the study.

### Visit Schedule, Randomization, Intervention, and Masking

The visit schedule of the DE/OL component of this study (Fig 1A) was designed to evaluate ONL1204 at 3 escalating dose levels. After screening/baseline procedures, the first patient was to receive a single IVT injection of 50 μg of ONL1204 in the study eye. If no safety concerns were identified, patient 2 was to be treated ≥7 days after the first patient received treatment. In the absence of safety concerns, patient 3 was to be treated with the 100 μg dose of ONL1204 14 days after patient 2 received treatment, and patient 4 was to be treated with 100 μg of ONL1204 ≥7 days after patient 3. The final 2 patients in the DE/OL component followed a similar staggered design, with patient 5 scheduled to receive a single 200 μg IVT injection of ONL1204 in the study eye 14 days after patient 4, if no safety concerns were identified. Patient 6 received treatment with 200 μg of ONL1204 ≥7 days after patient 5. All patients in the DE/OL component underwent screening/baseline assessments and were to be followed for 24 weeks.

Patients were enrolled concurrently in the NHS phase of component 2 with the DE/OL component 1. The study design allowed for up to 30 patients to be enrolled in the NHS/T component. A retinal imaging reading center was utilized in this NHS/T portion of the study to determine patient eligibility. Patients and images were required to be evaluated by the Principal Investigator and the images by Doheny Image Reading Center in order to meet eligibility. Patients were followed for approximately 24 weeks as part of the NHS phase after screening/baseline assessments. Once the NHS phase was complete, patients were randomized to 1 of 2 dose levels of ONL1204 (50 μg or 200 μg) or a sham injection (i.e., no injection). Randomization of patients in the NHS/T component was planned to occur ≥14 days after the last patient was treated in the DE/OL study component, allowing time for a Safety Review Committee (SRC) to assess any safety concerns. The treatment phase of the NHS/T component of the study involved 2 IVT injections administered 12 weeks apart. Follow-up after the second treatment was for an additional 12 weeks, for a total estimated study participation of 48 weeks. Patients in the DE/OL component followed the visit schedule presented in Figure 1A and patients in the NHS/T component followed the visit schedule presented in Figure 1B.

In the DE/OL component of the study, patients were assigned to the study drug based on enrollment sequence, with the first 2 patients assigned to the 50 μg dose, the next 2 assigned the 100 μg dose, and the last 2 patients assigned to receive the 200 μg dose of ONL1204. In the NHS/T component, patients were to be enrolled and followed for 24 weeks prior to randomization by blocks into 1 of 3 treatment groups (group 1: 50 μg dose, group 2: 200 μg, group 3: sham) in a 1:1:1 ratio involving treatment with 2 IVT injections of ONL1204 or 2 sham injections (i.e., no injection).

The NHS/T component of the study was double-masked. The study sponsor was not masked to study treatment. Patients who were randomized to sham did not receive an actual IVT injection. Instead, the same procedures were carried out in relation to study drug preparation and the IVT procedure, except an empty syringe with no needle attached was pressed against the anesthetized conjunctival surface. Patients were instructed to direct their gaze away from the syringe.

### SRC

Study oversight was provided by an SRC comprising site investigators, the medical monitor, and a sponsor representative. The SRC reviewed safety data to assess for dose-limiting toxicity (DLT). Determination of DLT included consideration of ocular inflammation, sustained elevation of intraocular pressure (IOP), reduction in visual acuity, and any serious adverse event (SAE). Additional details regarding the SRC review meetings, dose-escalating stopping criteria, and factors involved in the determination of DLT are presented in the Supplemental Information.

### Outcome Measures

The primary endpoints of this study were the safety and tolerability of ONL1204 in patients with GA secondary to AMD. This was assessed in both the DE/OL component (Fig 1A) and in the NHS/T component (Fig 1B). Safety was assessed based on adverse events (AEs) reported during the conduct of the study and clinical evaluations, including BCVA, IOP, electroretinography (ERG), and ophthalmoscopy. Clinical laboratory evaluations were also used to assess safety. Secondary endpoints included analysis of FAF images, spectral-domain OCT, and BCVA by ETDRS letters read. Exploratory analyses included OCT angiography outputs to assess lesion border integrity of the choriocapillaris. Additional details on the safety, ERG, and imaging methodology are included in the Supplemental Information. Blood was drawn for clinical labs in both study components. In the NHS/T component, plasma was tested for ONL1204 levels at 1 week after each injection and at the end of the study.

### Statistical Analyses

Three populations were used for analysis of the study results. The safety population included all participants who received any experimental investigational product within the study (ONL1204 or Sham). The safety population was used for the summaries of all safety assessments. The intent-to-treat (ITT) population included all screened participants who signed the informed consent form and were not considered screen failures, regardless of whether they received study drug or not. The ITT population included all NHS patients who did not progress to the treatment phase. The modified ITT population included all participants who were randomized, received the study drug, had ≥1 postinjection assessment of ETDRS BCVA, and had no important exclusionary protocol deviations. The modified ITT population was used for all efficacy analyses, unless otherwise stated.

Demographic (age, sex, race, and ethnicity) and baseline information were summarized descriptively. Baseline ocular characteristics included GA lesion size by FAF (mm^2^), GA lesion size by OCT (mm^2^), BCVA baseline (ETDRS letter score), low-luminance BCVA (ETDRS letter score), and low-luminance deficit.

The AEs were collected at each visit, and treatment-emergent AEs (TEAEs) were recorded after the first administration of study medication (ONL1204 Ophthalmic Solution). Additional details on the analysis of AEs/TEAEs are included in the Supplemental Information.

Summary statistics were calculated for each parameter of the safety laboratory assessments and ERG results (baseline and postbaseline visits). In addition, summaries included the change from baseline values at each scheduled postbaseline visit for each phase, as appropriate.

Best-corrected visual acuity was analyzed for safety and efficacy. The BCVA results were summarized for safety categorically as the number and percentage of patients with losses in letters read relative to baseline. A summary of IOP results was generated for study eye and fellow eye by visit. Slit lamp and indirect ophthalmoscopy grading assessments were summarized descriptively by treatment group and study visit for study eye and fellow eye.

Statistical modeling was done for each nonexploratory outcome type, including group differences between individual dose groups and sham, in addition to the combination of ONL1204 50 μg and 200 μg compared to sham. The change in area of GA and the rate of change of GA area were the areas of primary focus for the efficacy investigation. Additionally, mean change in BCVA and low-luminance visual acuity and low-luminance deficit were assessed. Comparisons against sham-treated eyes and fellow eyes were utilized, as well as changes from the NHS period to the treatment period in a given cohort.

For the rate of change analysis, a random effects coefficient regression model was used to compare the slopes of the treatment groups from baseline to the end of the study. This analysis used the continuous variable of study day as the time component. The model used an unstructured covariance structure and included a random intercept and slopes for study day and treatment, analyzed at the subject level to account for within-subject variability over time, as well as baseline imbalances. Due to the early-phase nature of the study and the generally descriptive nature of the study, given the small sample size, multiple comparisons and adjustments for multiplicity were not performed on any endpoint.

Other analyses were done using a mixed model for repeated measures with fixed effects of treatment, visit, and the interaction term of visit and treatment. The baseline value was included as a covariate. The model used an unstructured covariance structure and was analyzed at the subject level to account for within-subject variability over time, as well as baseline imbalances. Within-group analyses to evaluate change from baseline were performed using paired t-tests.

Missing data for all endpoints were assumed to be missing at random, and thus no imputation was performed. Given the mixed modeling methodology, the missing data were assumed to be accounted for by following the trend of their treatment group.

The number of patients was selected empirically for this phase Ib study. No formal sample size calculation was made, given the pilot nature of this study. All statistical analyses were conducted using SAS version 8.3 or higher (SAS Institute Inc).

## Results

**Figure 2:**
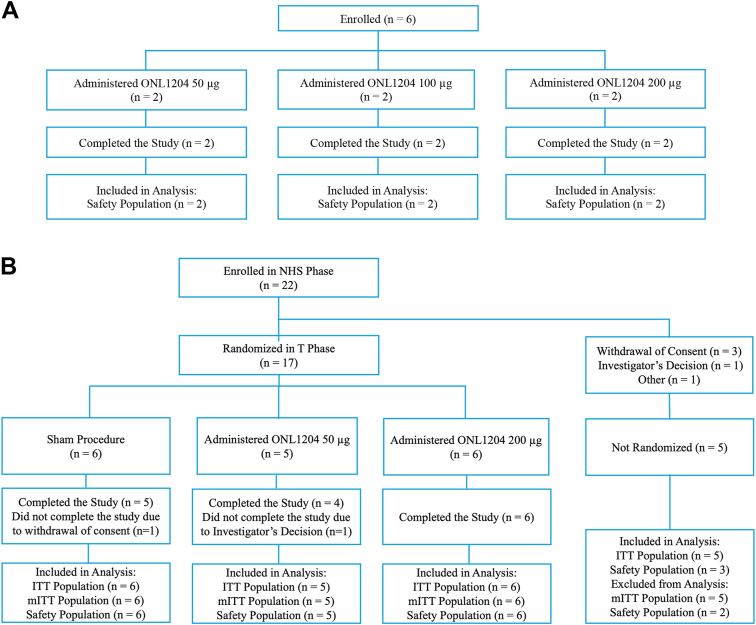
Patient flow diagram for the dose-escalation/open-label component **(A)** and the NHS/T component **(B)**. ITT = intent-to-treat; mITT = modified intent-to-treat; NHS = natural history; T = treatment.

A total of 55 patients were screened for eligibility, and 28 patients met eligibility criteria and were enrolled, including 6 patients in the DE/OL component and 22 in the NHS/T component. Six patients who were enrolled and completed the DE/OL component of the study each received a single injection of ONL1204 at 50, 100, or 200 μg (nonrandomized; n = 2 per dose). All patients who were enrolled in the DE/OL were included in the safety population (Fig 2A). A total of 22 patients were enrolled in the NHS component of the study, of whom 17 were randomized into the treatment phase, while 5 patients withdrew prior to randomization. The treatment phase of the study included the 17 patients who continued beyond the NHS phase of the study, with 6 patients randomized to sham, 5 patients to ONL1204 50 μg, and 6 patients to ONL1204 200 μg. Overall, 15 patients completed the treatment phase of the study (5 in the sham group, 4 in the 50 μg group, and 6 in the 200 μg group). One patient in the sham group withdrew consent after receiving only 1 sham treatment and did not complete the study; 1 patient in the ONL1204 50 μg group withdrew based on the investigator's decision after the second injection, due to general health issues, and did not complete the study (Fig 2B). Five (83.3%) patients in the sham group, 4 (80.0%) patients in the 50 μg ONL1204 treatment group, and 5 (83.3%) patients in the 200 μg ONL1204 group received both treatments, as planned. One patient in the 200 μg ONL1204 group received only the first injection of study drug due to an AE of vitreous floaters and completed the study.

The mean age of patients enrolled in the DE/OL population was 78.3 years. Five of 6 (88.3%) patients were White, and 4 of 6 (66.7%) patients were women. The mean age of patients in the NHS/T group was 77.6 years. All patients were White and 18 of 22 (81.8%) were women. The demographic and baseline ocular characteristics of the study population are presented by treatment group and study component in Table 1.

**Table 1:** Demographics and Baseline Ocular Characteristics

Dose-Escalation/Open-Label (DE/OL) Component					
Parameter	Statistic	ONL1204 50 μg (n = 2)	ONL1204 100 μg (n = 2)	ONL1204 200 μg (n = 2)	All Patients (n = 6)
Age (yrs)	Mean (SD)	82 (5.7)	79.5 (3.5)	73.5 (9.2)	78.3 (6.4)
	Range	78–86	77–82	67–80	67–86
Sex, n (%)	Female	2 (100.0%)	2 (100.0%)	0 (0.0%)	4 (66.7%)
	Male	0 (0.0%)	0 (0.0%)	2 (100.0%)	2 (33.3%)
Race, n (%)	White	2 (100.0%)	2 (100.0%)	1 (50.0%)	5 (83.3%)
	Asian	0 (0.0%)	0 (0.0%)	1 (50.0%)	1 (16.7%)
Ethnicity, n (%)	Not Hispanic or Latino	2 (100.0%)	2 (100.0%)	2 (100.0%)	6 (100.0%)
BCVA (ETDRS letters)	Mean (SD)	24.5 (2.1)	12.5 (2.1)	29.0 (14.1)	22.0 (10.0)
	Range	23–26	11–14	19–39	11–39

**Natural history/treatment (NHS/T) component**					

Parameter	Statistic	Sham (n = 6)	ONL1204 50 μg (n = 5)	ONL1204 200 μg (n = 6)	All Patients (n = 22)

Age (yrs)	Mean (SD)	75.5 (8.3)	77.6 (12.6)	78.8 (4.3)	77.6 (7.5)
	Range	67–91	63–95	72–85	63–95
Sex, n (%)	Female	5 (83.3%)	2 (40.0%)	6 (100.0%)	18 (81.8%)
	Male	1 (16.7%)	3 (60.0%)	0 (0.0%)	4 (18.2%)
Race, n (%)	White	6 (100.0%)	5 (100.0%)	6 (100.0%)	22 (100.0%)
Ethnicity, n (%)	Not Hispanic or Latino	6 (100.0%)	5 (100.0%)	6 (100.0%)	21 (95.5%)
	Unknown	0 (0.0%)	0 (0.0%)	0 (0.0%)	1 (4.5%)
GA area by FAF (mm^2^)	Mean (SD)	12.9 (7.0)	12.9 (6.5)	9.7 (8.2)	11.0 (7.0)
	Range	2.5–18.8	5.5–20.4	2.3–25.7	2.3–25.7
GA area by OCT (mm2)	Mean (SD)	8.7 (6.8)	11.8 (5.1)	7.9 (3.0)	9.0 (4.6)
	Range	2.8–16.1	6.6–17.3	3.7–10.9	2.8–17.3
BCVA (ETDRS letters)	Mean (SD)	60.2 (12.2)	54.6 (12.1)	45.7 (17.9)	54.3 (14.9)
	Range	38–75	34–63	26–75	26–79
LLBCVA (ETDRS letters)	Mean (SD)	27.2 (22.4)	9.8 (4.8)	29.2 (14.7)	23.7 (15.5)
	Range	7–65	3–14	15–52	3–65

BCVA = best-corrected visual acuity; GA = geographic atrophy; FAF = fundus autofluorescence; LLBCVA = low-luminance best-corrected visual acuity; n = number; SD = standard deviation.

### Safety

The most frequently occurring ophthalmic TEAEs reported in this study included conjunctival hemorrhage, vitreous floaters, eye pain, foreign body sensation in the eyes, and punctate keratitis. A summary of the most common (≥2) TEAEs associated with ONL1204-treated patients in the safety population is presented in Table 2. The majority of TEAEs that occurred during the study, including all ophthalmic TEAEs, were mild or moderate in severity. A total of 4 SAEs were reported during the study, including 2 serious TEAEs and 2 pretreatment SAEs. All SAEs were nonophthalmic in nature (atrial fibrillation, cellulitis, malignant neoplasm, and a fall), and none were deemed related to the study drug. A summary of all TEAEs reported associated with the study eye is presented in Table S3 located in the Supplemental Information (available at www.ophthalmologyscience.org).

**Table 2:** Summary of TEAEs

TEAE Summary	
Total TEAEs	Number of TEAEs; Number of Patients (%)
TEAEs in patients treated with ONL1204	51; 14 (82.4%)
TEAEs in the sham group	24; 6 (100.0%)
Ophthalmic TEAEs - study eye	
TEAEs in patients treated with ONL1204	23; 12 (70.6%)
TEAEs in the sham group	9; 3 (50.0%)
Ophthalmic TEAEs - fellow eye	
TEAEs in patients treated with ONL1204	12; 5 (29.4%)
TEAEs in the sham group	4; 3 (50.0%)
Nonophthalmic TEAEs	
TEAEs in patients treated with ONL1204	16; 10 (58.8%)
TEAEs in the sham group	11; 5 (83.3%)

Ophthalmic TEAEs Reported in ≥2 ONL1204-Treated Patients (Safety Population)					
Preferred Term	Sham (n = 6) n (%) m	ONL1204 50 μg (n = 7) n (%) m	ONL1204 100 μg (n = 2) n (%) m	ONL1204 200 μg (n = 8) n (%) m	All ONL1204 (n = 17) n (%) m
Conjunctival hemorrhage	0	1 (14.3) 2	0	2 (25.0) 2	3 (17.6) 4
Vitreous floaters	0	2 (28.6) 2	0	1 (12.5) 1	3 (17.6) 3
Eye pain	1 (16.7) 1	1 (14.3) 1	0	1 (12.5) 1	2 (11.8) 2
Foreign body sensation in eyes	2 (33.3) 2	0	0	2 (25.0) 2	2 (11.8) 2
Punctate keratitis	1 (16.7) 1	1 (14.3) 1	0	1 (12.5) 1	2 (11.8) 2

Nonophthalmic Events					
Urinary tract infections	0	1 (14.3) 1	0	1 (12.5) 1	2 (11.8) 2
Dizziness	0	2 (28.6) 2	0	0	2 (11.8) 2

m = number of occurrences; n = number of patients; TEAE = treatment-emergent adverse event.

Treatment-emergent adverse events are defined as adverse events that occurred after the first administration of study medication. If a patient had multiple occurrences of a TEAE, the patient is presented only once in the patient count (n) column for a given preferred term. Occurrences are counted each time in the mentions/occurrence (m) column. Percentages are calculated (the denominator used for the calculation) based on the number of patients in the safety population in each treatment group (n).

There were 3 TEAEs in 2 patients (IOP increased and vitreous floaters in 1 patient and open-angle glaucoma in the other) that were considered related to the study drug—all occurring in the study eye and in patients in the ONL1204 200 μg treatment group. All 3 of these events were considered ongoing at the end of the study. Additional details on TEAEs are included in the Supplemental Information. The remaining AEs were deemed unrelated to the study drug. There were 2 procedure-related AEs. Both were in study eyes, mild and resolved. One was foreign body sensation, and the other was punctate keratitis.

No safety concerns based upon an analysis of ERG, BCVA, mean IOP change, and ophthalmoscopy assessment or clinical laboratory evaluations that would preclude progression to a larger trial of ONL1204 Ophthalmic Solution were identified for any dose tested. ONL1204 was undetectable in plasma (lower limit of quantitation: 1 ng/mL), indicating that there was no measurable systemic exposure to ONL1204 after up to 2 IVT injections. There were no cases of ERG changes attributable to treatment with ONL1204 during the study; 1 patient in the DE/OL component showed a decrease of >40% in rod b-wave amplitude at visits 4 and 5, and an ad hoc SRC meeting was held to discuss the findings. This independent review concluded that this reduction was primarily an artifact due to high baseline recording. There were neither DLTs nor new cases of choroidal neovascularization observed in this study in either eye.

### Efficacy

While GA lesion areas were measured using both FAF and OCT images, the FAF approach was more reliable in this dataset because the macular area assessed was greater with FAF imaging, with incomplete capture of GA lesion area by OCT analysis. Geographic atrophy lesion sizes were quantified using both the absolute value (mm^2^) and the square root transformation (mm). While the intent of the square root transformation was to minimize variance of the values, no additional information was gleaned from the square root transformed data, and therefore the data presented here focuses on the absolute GA areas as assessed by FAF.

**Figure 3:**
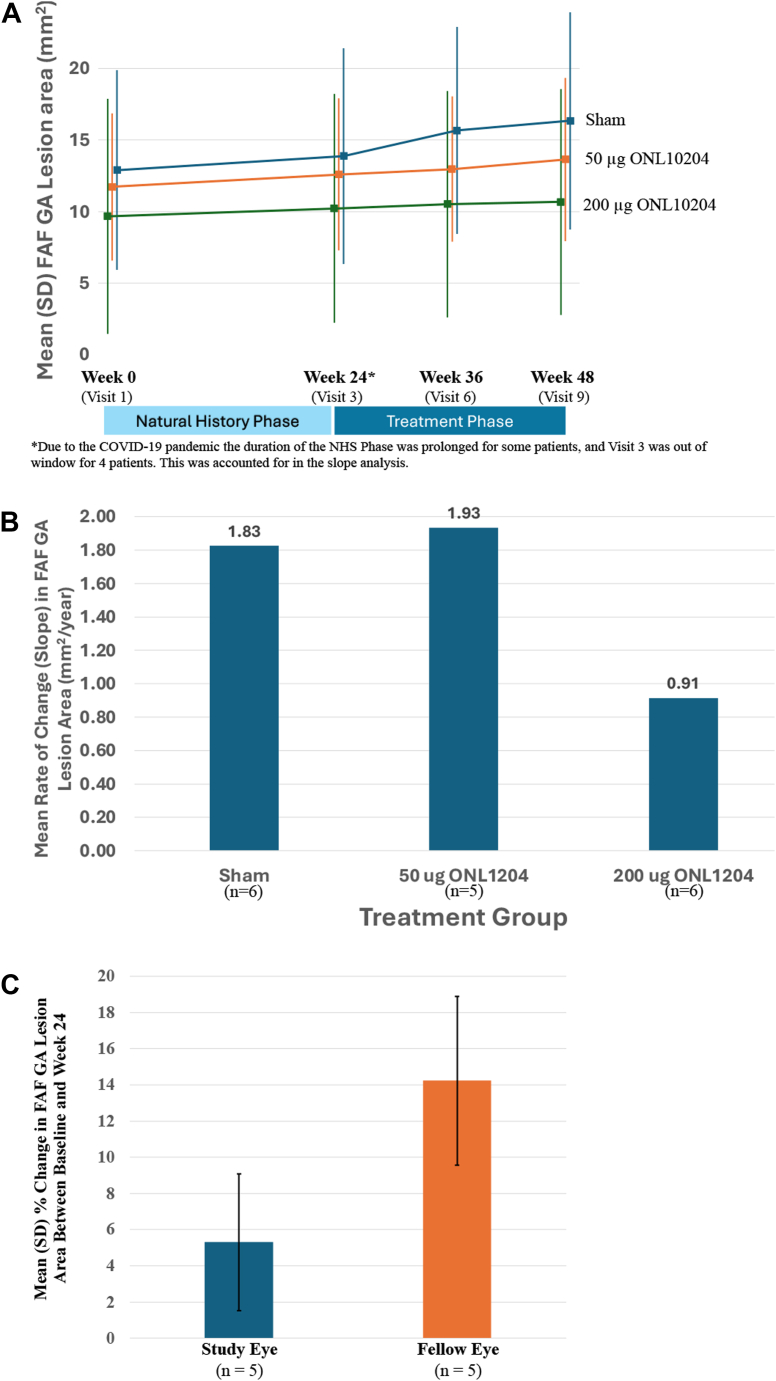
Geographic atrophy lesion area. Mean (SD) raw FAF GA lesion areas in the study eye are presented by treatment group and visit across the NHS/T component **(A)**. The mean rate of change (slope) in FAF GA lesion area by treatment group in the study eye in the mITT population is presented from the baseline of the treatment phase until the end of study **(B)**. Lesion area of mm^2^/year annualized based on calculation of mm^2^/day. The mean (SD) percent change in FAF GA lesion area from baseline to week 24 in the study eye and the fellow eye is presented for the DE/OL component of the study **(C)**. Data presented for patients in the DE/OL group with gradable lesions only. COVID-19 = coronavirus disease 2019; DE = dose-escalation; FAF = fundus autofluorescence; GA = geographic atrophy; mITT = modified intent-to-treat; NHS = natural history; OL = open-label; T = treatment; SD = standard deviation.

Mean GA lesion area assessed by FAF is presented by treatment group and study visit for the NHS/T component in Figure 3A. The mean rate of lesion area growth (slope) was similar during the NHS phase (visit 1–visit 3) across treatment groups. In the treatment phase (visit 3–visit 9), the rate of growth (slope) was numerically reduced in 200 μg ONL1204-treated eyes, while the rate of growth of the lesion areas remained similar to the NHS phase for the 50 μg ONL1204-treated eyes and sham group (Fig 3B). The slope of lesion growth (using a per day rate of change) during the treatment phase based on these timepoints for sham, ONL1204 50 μg, and ONL1204 200 μg were 0.0050 mm^2^ per day, 0.0053 mm^2^ per day, and 0.0025 mm^2^ per day, respectively. The difference in slopes from the 200 μg ONL1204 treated patients during the treatment phase compared to sham-treated eyes in a paired analysis was 50% lower compared with sham-treated patients; however, this difference did not reach statistical significance (*P* = 0.289). In the DE/OL component of the study, mean GA lesion area was assessed in the study and fellow eyes for comparison. Comparisons in growth rate were able to be made for 5 patients (83.3%) with bilateral gradable lesions. Of these 5 patients, the mean GA lesion area in the study eyes for the DE/OL component was 14.56 mm^2^, and the mean GA area was 11.01 mm^2^ for the fellow eyes. For these 5 patients with bilaterally gradable lesions, the mean percent change in the GA lesion area for the study eye was an increase of 5.3%, while the mean percent change in the fellow eyes for these same patients was an increase of 14.2% (Fig 3C).

**Figure 4:**
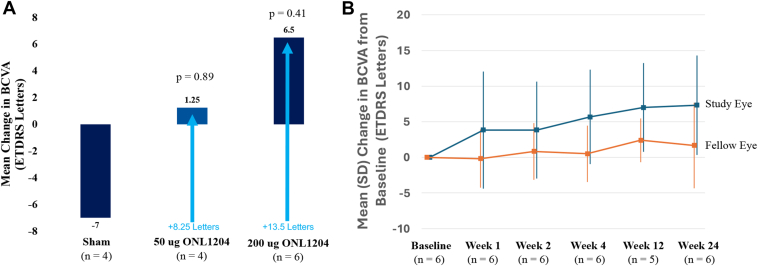
Visual acuity outcomes. The mean change in BCVA (ETDRS letters) in the study eye is presented across treatment groups during the NHS/T component of the study **(A)**. The bars reflect the change in BCVA observed from the baseline of the NHS phase to the end of the study (week 24 of the treatment phase). The arrows present the ETDRS letter difference between the ONL1204 treatment groups and the sham control group at the end of the study. The mean (SD) change from baseline in BCVA (ETDRS letters) in the study eye and fellow eye is presented by visit in the DE/OL component of the study **(B)**. BCVA = best-corrected visual acuity; DE = dose-escalation; NHS = natural history; OL = open-label; T = treatment; SD = standard deviation.

Mean BCVA (ETDRS letters) generally decreased during the NHS phase (visit 1 to visit 3) across all treatment groups. Mean BCVA changes from baseline during the treatment phase compared with the NHS phase were –7.00 ETDRS letters for sham participants, +1.25 ETDRS letters for ONL1204 50 μg participants, and +6.50 ETDRS letters for ONL1204 200 μg participants, where a positive value means better BCVA compared with baseline. This differential between the ONL1204 50 μg group and sham group at the end of the study was +8.25 ETDRS letters, and this same differential for the ONL1204 200 μg group relative to the sham group was +13.5 ETDRS letters (Fig 4A).

Mean change from baseline in BCVA in the study eye and fellow eye by visit in the DE/OL component of the study is presented in Figure 4B. Mean BCVA at baseline for the study and fellow eyes in the DE/OL component was 23.0 and 66.8 ETDRS letters, respectively; mean BCVA at week 24 was 30.3 and 68.5 ETDRS letters, indicating a mean change (improvement) of 7.3 and 1.6 letters for the study eyes and fellow eyes, respectively.

Similar to BCVA, favorable changes were observed with respect to assessments of low-luminance visual acuity during the treatment phase compared with the NHS phase for the ONL1204 200 μg dose group. The mean difference in changes from baseline (visit 9 [end of study] to visit 3 [treatment phase baseline] compared with visit 3 [end of NHS] to visit 1 [NHS baseline]) was –0.33 ETDRS letters for the sham group, –4.00 ETDRS letters for the ONL1204 50 μg group (*P* = 0.215 vs. sham), and +9.00 ETDRS letters in the ONL1204 200 μg group (*P* = 0.0467 vs. sham).

Due to a small number of usable data points available for the OCT angiography analysis, it was not possible to draw meaningful conclusions on changes in choriocapillaris flow deficit based on the OCT angiography data from this study.

## Discussion

The primary purpose of this multicenter phase Ib study was to evaluate the safety and tolerability of ONL1204. This is the first evaluation of ONL1204, a novel inhibitor of Fas-mediated cell death, for the treatment of patients with GA secondary to AMD, and as such it was not designed to identify rare AEs. The administration of ONL1204 via IVT injection was generally safe and well-tolerated at all doses tested in both the DE/OL component and in the NHS/T component of this study. There was no evidence of DLT observed after a single IVT injection in the DE/OL component or after 2 injections of ONL1204 administered 3 months apart in the NHS/T component. A favorable safety and tolerability profile was observed, with all the TEAEs being classified as mild or moderate in severity. The safety profile will be monitored carefully in subsequent studies, particularly with respect to the TEAEs that were designated as related to study treatment, including increased IOP (1 event in 1 patient), open-angle glaucoma (1 event in 1 patient), and vitreous floaters (1 event in 1 patient).

This study was not powered for efficacy; however, the secondary efficacy endpoints did show potential signals of reduction (slowing) in the progression of GA lesion area among eyes treated with ONL1204. Directionally favorable changes in ETDRS BCVA and low-luminance visual acuity were also observed among eyes treated with the highest dose of ONL1204. These results may indicate potential for benefit in BCVA in eyes with GA with ONL1204 treatment. Although the DE/OL component was included primarily for safety, the GA lesions in study eyes appeared to grow at roughly one-third the rate as in fellow eyes. Although there may have been a drug effect leading to this difference, an important caveat is that GA lesions can grow in a heterogeneous manner, and baseline morphology and size can potentially affect growth rates. It is possible that these larger lesions in the study eyes naturally progressed at a slower rate than the smaller lesions of fellow eyes.^13^^,^^14^ These are very small numbers of eyes studied over a relatively short (6-month) duration of time, further suggesting these results be interpreted cautiously. The GA area data from this group of participants has been included for completeness, as this information too may help guide the design of larger, well-controlled studies.

The results of this study suggest that the novel mechanism of action of ONL1204 as a small peptide acting to inhibit the interaction of the Fas receptor with Fas ligand may offer new opportunities to slow the progression of GA associated with AMD. The Fas signaling pathway is directly related to the regulation of programmed cell death and also serves as an upstream mediator for a range of inflammatory pathways involved in the immune response, including the release of cytokines and chemokines and activation of the complement system.^10^^,^^11^ These early insights into the potential efficacy of ONL1204 to protect retinal cells from Fas-mediated apoptosis in this condition warrant further clinical evaluation.

The main limitations of this study include its sample size of 28 patients and follow-up limited to 6 months. The primary objective of the study was achieved as assessed by the evaluation of the safety and tolerability of multiple concentrations of ONL1204 delivered by IVT injection. Of note, there were no new cases of choroidal neovascularization identified in treated patients. This study was not designed to be powered to detect statistically significant differences in the secondary efficacy endpoints; however, the favorable safety profile of ONL1204 observed, combined with directionally positive efficacy signals, provides important insights that are guiding the design of future clinical trials among patients with GA.

An upcoming phase II study will address the limitations of the present study in terms of sample size and a deeper exploration of optimized dosing. It will also utilize more treatments and provide for a longer follow-up period after dosing to further assess the potential role of ONL1204 in the treatment of GA.
