# Supplementary material for: ONL1204 for the Treatment of Geographic Atrophy: Phase Ib Study Evaluating Safety, Tolerability, and Efficacy
Source: Ophthalmol Sci. 2025 Oct 3;6(1):100954. doi: 10.1016/j.xops.2025.100954 (PMC12613103; doi:10.1016/j.xops.2025.100954)
Supplement: Supplemental Information [file mmc1.pdf]

Supplemental Information:

Additional Details Regarding Safety and Efficacy Assessments

Medical History

Medical history was coded using the Medical Dictionary for Regulatory Activities (MedDRA) Version 23.1 (MedDRA MSSO, Herndon, VA, USA).

Adverse Events (AEs)

The AEs were collected from the time of obtaining consent and at each visit until patients exited the study. Treatment-emergent AEs (TEAEs) were defined as events that occurred or worsened after the first administration of study medication (ONL1204 Ophthalmic Solution). Summary analyses of AEs were performed only for those events that were considered TEAEs. TEAEs were summarized by incidence rates. Ophthalmic and non-ophthalmic AEs were recorded and analyzed separately. TEAEs by severity and relationship to study drug were also summarized. Ophthalmic AEs were split into study eye and fellow eye events. Events reported in both eyes were included in the study eye and fellow eye analysis.

Electroretinography (ERG)

ERG (RET*eval*®, LKC Technologies, Gaithersburg, MD) was included as a safety assessment in this study to support the primary endpoint of safety and tolerability. Prespecified criteria for follow up evaluation of patients with ERG changes were established to determine repeatability of the findings. If 2 or more patients showed repeatable change in their ERG, results were presented to the SRC for a decision regarding relation to ONL1204 and subsequent action.

Imaging Methodology

All acquisition protocols utilized industry standard approaches, and site certification was required. Details regarding the make and models of equipment, hardware and software requirements and image submission requirements are listed in the tables below.

Equipment and Software Requirements

Modality	Make and Models	Hardware and Software Requirements
**Optical Coherence Tomography (OCT)**	Heidelberg Engineering<br>• Spectralis OCT or OCT Plus (with or without BluePeak)<br>• Spectralis FA+OCT<br>• Spectralis HRA+OCT	Version: 5.6 or higher<br>• HRA / Spectralis Family Acquisition Module<br>• HRA / Spectralis Viewing Module
**Optical Coherence Tomography Angiography (OCTA)**	Heidelberg Engineering<br>• Spectralis OCT or OCT Plus (with or without BluePeak)<br>• Spectralis FA+OCT<br>• Spectralis HRA+OCT	Version: 6.9 or higher<br>• HRA / Spectralis Family Acquisition Module<br>• HRA / Spectralis Viewing Module<br>• With the OCTA module
**Fundus Autofluorescence (FAF)**	Heidelberg Engineering<br>• HRA2<br>• Spectralis OCT or OCT Plus (With BluePeak)<br>• Spectralis HRA<br>• Spectralis FA+OCT<br>• Spectralis HRA+OCT	Version: 5.6 or higher<br>• HRA2 systems<br>• Spectralis systems
**Color Fundus Photography (FP) and Fluorescein Angiography (FA)**	Standard quality digital fundus camera with ability to export images.	Field of View (FOV): 30° to 60°

Capture Study Visit Images – Imaging Requirements.

Modality	Image Submission Requirements
**Optical Coherence Tomography (OCT)**	Volume scans (20°x20°, High Resolution, 97 sections, Automatic Real Time (ART) Mean: ≥4 Frames)
**Optical Coherence Tomography Angiography (OCTA)**	Volume scans, OCTA 10°x10°<br>(3mm x 3mm); Volume scans, OCTA 20°x20° (6mm x 6mm)
**Fundus Autofluorescence (FAF)**	• IR image – Field 2 (centered on macula)<br>• Blue autofluorescence (BAF) image – Fields 1M, 2, and 3M
**Color Fundus Photography (FP) and Fluorescein Angiography (FA)**	Modified 3-standard fields: Field 2 Red Free, Field 1M, Field 2, Field 3M
