# Supplementary material for: ONL1204 for the Treatment of Geographic Atrophy: Phase Ib Study Evaluating Safety, Tolerability, and Efficacy
Source: Ophthalmol Sci. 2025 Oct 3;6(1):100954. doi: 10.1016/j.xops.2025.100954 (PMC12613103; doi:10.1016/j.xops.2025.100954)
Supplement: Table S3 [file mmc2.pdf]

Table 3. Ophthalmic Treatment-emergent Adverse Events (TEAEs)

Ophthalmic TEAEs Reported in ONL1204-treated Patients (Study Eye: Enrolled Population)				
**Preferred Term**	**Occurrence m (n)**	**Treatment**	**Severity**	**Relatedness**
Blepharitis	1 (1)	ONL1204 200 ug	Mild	Not related
Conjunctival hemorrhage	2 (1)	ONL1204 50 ug	Mild	Not related
	2 (2)	ONL1204 200 ug	Mild	Not related
Dry eye	1 (1)	ONL1204 50 ug	Moderate	Not related
Eye irritation	1 (1)	ONL1204 200 ug	Moderate	Not related
Eye pain	1 (1)	ONL1204 50 ug	Mild	Not related
	1 (1)	ONL1204 200 ug	Moderate	Not related
Foreign body sensation eye	1 (1)	ONL1204 200 ug	Mild	Related to study drug administration; Related to other study procedure
	1 (1)	ONL1204 200 ug	Mild	Not related
Intraocular pressure increased	1 (1)	ONL1204 200 ug	Moderate	Related to Study Drug
Open angle glaucoma	1 (1)	ONL1204 200 ug	Moderate	Related to Study Drug
Photopsia	1 (1)	ONL1204 50 ug	Mild	Not related
Punctate keratitis	1 (1)	ONL1204 200 ug	Mild	Related to Other Study Procedure
	1 (1)	ONL1204 50 ug	Moderate	Not related
Retinal hemorrhage	1 (1)	ONL1204 100 ug	Mild	Not related
Vitreous degeneration	1 (1)	ONL1204 50 ug	Mild	Not related
Vitreous detachment	1 (1)	ONL1204 200 ug	Mild	Not related
Visual field defect	1 (1)	ONL1204 50 ug	Mild	Not related
Visual impairment	1 (1)	ONL1204 50 ug	Mild	Not related
Vitreous floater	2 (2)	ONL1204 50 ug	Mild	Not related
	1 (1)	ONL1204 200 ug	Moderate	Related to Study Drug

Abbreviations: m = number of occurrences; n = number of patients; TEAE = Treatment-emergent Adverse Events
