# Supplementary material for: ONL1204 for the Treatment of Geographic Atrophy: Phase Ib Study Evaluating Safety, Tolerability, and Efficacy
Source: Ophthalmol Sci. 2025 Oct 3;6(1):100954. doi: 10.1016/j.xops.2025.100954 (PMC12613103; doi:10.1016/j.xops.2025.100954)
Supplement: Supplemental Information [file mmc3.pdf]

Supplemental Information:

Dose Escalation/Open Label Study Component

Inclusion Criteria

Patients who met all of the following inclusion criteria at Screening were eligible to participate:

1. Males and females, ≥ 55 years old
2. Willing and able to give informed consent and attend study visits
3. GA secondary to AMD in the study eye
4. ETDRS BCVA 20/100 to Count Fingers (Snellen equivalent) in the study eye
5. Study eye must have been the poorer seeing eye, or the 2 eyes must have been equivalent, based on ETDRS BCVA
6. Female patients must have been:
    a. Women of non-childbearing potential
    b. or Women of childbearing potential (WOCBP) with a negative pregnancy test at Screening and willing to use permissible methods of contraception for the duration of the study
7. Males with female partners of childbearing potential must have agreed to use permissible methods of contraception and must have agreed to refrain from donating sperm for the duration of the study.

Exclusion Criteria

Patients who met any of the following exclusion criteria at Screening were not eligible to participate:

1. GA in the study eye due to causes other than AMD
2. Participation in other ophthalmic clinical trials or use of any other investigational drugs or devices in study eye or systemically for 6 months prior to enrollment, or anticipated participation in other ophthalmic clinical trials or use of any other investigational drugs or devices in study eye or systemically during the study period
3. Intraocular inflammation in the study eye
4. Ocular or periocular infection in the study eye
5. Media opacity that would have limited Baseline visual acuity or clinical visualization of the retina at Baseline in the study eye
6. Prior history of systemic use of pentosan polysulfate sodium (trade name Elmiron®)
7. Any ocular or systemic condition that in the opinion of the Investigator made the patient unsuitable for treatment with an investigational agent or that would have compromised the safety and efficacy assessments of the trial.

Natural History Study/Treatment Study Component

Inclusion Criteria

Patients who met all of the following inclusion criteria at Screening were eligible to participate:

1. Males and females, ≥ 55 years old
2. Willing and able to give informed consent and attend study visits
3. Bilateral GA secondary to AMD without choroidal neovascularization in either eye as determined by the reading center and Investigator
4. ETDRS BCVA 20/400 (Snellen equivalent) or better in both eyes
5. GA ≥ 1 disc area (DA) (equivalent to ≥ 2.5 mm^2) in the study eye as determined by the reading center
6. If GA in study eye was multifocal, at least one focal lesion must have had a DA of ≥ 1.25 mm^2 as determined by the reading center
7. Entire GA area must have been visible within the standard FAF field of view in the study eye as determined by the reading center
8. Presence of banded or diffuse hyperautofluoresence adjacent to GA lesion in the study eye as determined by the reading center
9. Female patients must have been:
    a. Women of non-childbearing potential, or
    b. WOCBP with a negative pregnancy test at Screening and willing to use permissible methods of contraception for the duration of the study
10. Males with female partners of childbearing potential must have agreed to use permissible methods of contraception and must have agreed to refrain from donating sperm for the duration of the study.

Exclusion Criteria

Patients who met any of the following exclusion criteria at Screening were not eligible to participate:

1. GA in either eye due to causes other than AMD as determined by the reading center and Investigator
2. Participation in other ophthalmic clinical trials or use of any other investigational drugs or devices in study eye or systemically for 6 months prior to enrollment, or anticipated participation in other ophthalmic clinical trials or use of any other investigational drugs or devices in study eye or systemically during the study period
3. Intraocular inflammation in the study eye
4. Ocular or periocular infection in the study eye
5. Media opacity that would limit Baseline visual acuity or clinical visualization of the retina at Baseline

6. Hyperautofluoresence adjacent to GA lesion in study eye that was focal only as determined by the reading center
7. Previous IVT treatment, history of retinal surgery, or other retinal therapeutic procedures in the study eye
8. Systemic immunosuppression that could interfere with retinal and cytokine expression including but not limited to glucocorticoids (eg, oral prednisone or dexamethasone); antimetabolites (eg, methotrexate, mycophenolate mofetil, and azathioprine); T-cell inhibitors (eg, cyclosporine, tacrolimus, sirolimus); alkylating agents (eg, cyclophosphamide and chlorambucil); and biologic agents (eg, tumor necrosis factor inhibitors, interferons, lymphocyte inhibitors, and interleukin inhibitors)
9. Prior history of systemic use of pentosan polysulfate sodium (trade name Elmiron®)
10. Any ocular or systemic condition that in the opinion of the Investigator made the patient unsuitable for treatment with an investigational agent or that would have compromised the safety and efficacy assessments of the trial
11. An unwillingness to elect to either a) use Age-related Eye Disease Study 2 (AREDS2) formula nutraceutical therapy for the duration of the study or b) choose not to use such therapy for the duration of the study
