# Supplementary material for: ONL1204 for the Treatment of Geographic Atrophy: Phase Ib Study Evaluating Safety, Tolerability, and Efficacy
Source: Ophthalmol Sci. 2025 Oct 3;6(1):100954. doi: 10.1016/j.xops.2025.100954 (PMC12613103; doi:10.1016/j.xops.2025.100954)
Supplement: Supplemental Information [file mmc4.pdf]

Supplemental Information:

Dose-limiting Toxicity

Oversight was provided by a Safety Review Committee (SRC). The SRC reviewed safety data from available patients to determine if there was any dose-limiting toxicity. The SRC reviewed safety data at a minimum of 14 days after the sixth dose-escalation patient (the 2nd patient at the 200 ug dose level) was treated, or at 4 weeks prior to randomization (whichever was later in the course of the study). The SRC was also able to meet on an ad hoc basis, as needed.

Dose escalation Stopping Criteria

Dose-limiting toxicity, if observed, would be declared by the SRC, based on their review of the relevant clinical information. Determination of DLT included consideration of the following events:

1. Ocular inflammation increases by 3 units from pre-injection on the Standardization of Uveitis Nomenclature (SUN) grading scale for aqueous cell or vitreous haze, secondary to inflammation and not the drug formulation itself that persisted through post-injection Day 14;
2. Sustained elevation of IOP, characterized as > 30 mmHg for 7 consecutive days, post-injection of ONL1204, within the first 14 days following ONL1204 injection, despite pharmacologic therapy;
3. Reduction in visual acuity from Baseline after injection of ONL1204 that, in the opinion of the Investigator, was likely due to the study drug and resulted in:
   a. At least a 6-line (30 letter) or 0.6 logarithm of the Minimum Angle of Resolution (logMAR) decrease within 14 days of the injection of ONL1204;
   b. At least a 3-line (15 letter) or 0.3 logMAR decrease within the first 7 days following an injection of ONL1204 that was still present at the next scheduled visit; or
   c. Decrease in visual acuity from Baseline to light perception, or no light perception.

   Note: If a patient could no longer read letters on the ETDRS chart, counts fingers vision was to be treated as 2.0 logMAR, hand motions vison was to be treated as 3.0 logMAR, light perception as 3.9 logMAR, and no light perception as 4.0 logMAR.
4. Any serious adverse event (SAE) that occurred within the first 14 days following an ONL1204 injection that, in the opinion of the Investigator, was related to ONL1204.
