# Supplementary material for: ONL1204 for the Treatment of Geographic Atrophy: Phase Ib Study Evaluating Safety, Tolerability, and Efficacy
Source: Ophthalmol Sci. 2025 Oct 3;6(1):100954. doi: 10.1016/j.xops.2025.100954 (PMC12613103; doi:10.1016/j.xops.2025.100954)
Supplement: Supplemental Information [file mmc5.pdf]

Supplemental Information:

Additional Details Pertaining to TEAEs Classified as Related to Study Drug

There were 3 TEAEs that occurred in 2 patients that were considered related to the study drug. All 3 of these TEAEs occurred in the study eye and in patients in the ONL1204 200 μg treatment group. Two of the TEAEs were experienced by one patient. The first was an increase in IOP (moderate in severity), with the event starting on Day 8 of the Treatment phase. The IOP in the study eye increased from a Treatment Phase Baseline of 11 mmHg to as high as 45 mmHg at Week 4 (Visit 5). The patient was treated with brimonidine tartrate, brinzolamide, latanoprost, latanoprost/timolol maleate and selective laser trabeculoplasty. The patient discontinued the trial. The second was vitreous floaters (moderate in severity) in the same patient, with the event starting on Day 64 of the treatment phase. The event was treated by the site PI with phenylephrine hydrochloride and prednisolone acetate and the scheduled second injection of study drug was not administered. Finally, open-angle glaucoma (moderate in severity) was documented in a different patient receiving ONL1204 200 μg in the DE/OL cohort, with the event starting on Day 162 (Month 6 visit). The patient was treated with brimonidine tartrate, brinzolamide and latanoprost;timolol maleate. All three of these events were considered ongoing at the end of the study.
